# Synergistic Effect of Nanoparticles: Enhanced Mechanical and Corrosion Protection Properties of Epoxy Coatings Incorporated with SiO_2_ and ZrO_2_

**DOI:** 10.3390/polym15143100

**Published:** 2023-07-20

**Authors:** Ubair Abdus Samad, Mohammad Asif Alam, Hany S. Abdo, Arafat Anis, Saeed M. Al-Zahrani

**Affiliations:** 1 Center of Excellence for Research in Engineering Materials (CEREM), King Saud University, Riyadh 11421, Saudi Arabia; uabdussamad@ksu.edu.sa; 2 Mechanical Design and Materials Department, Faculty of Energy Engineering, Aswan University, Aswan 81521, Egypt; 3 Department of Chemistry, College of Science, King Saud University, P.O. Box 2455, Riyadh 11451, Saudi Arabia; aarfat@ksu.edu.sa; 4 SABIC Polymer Research Center (SPRC), Chemical Engineering Department, King Saud University, P.O. Box 800, Riyadh 11421, Saudi Arabia; szahrani@ksu.edu.sa

**Keywords:** adhesive coatings, synergistic effect, mechanical properties, nanoparticles

## Abstract

This research paper presents the fabrication of epoxy coatings along with the hybrid combination of SiO_2_ and ZrO_2_. The epoxy resin is incorporated with SiO_2_ as the primary pigment and ZrO_2_ as the synergist pigment. The study delves into the adhesion, barrier, and anti-corrosion properties of these coatings, enriched with silica and zirconium nanoparticles, and investigates their impact on the final properties of the epoxy coating. The epoxy resin, a Diglycidyl ether bisphenol-A (DGEBA) type, is cured with a polyamidoamine adduct-based curing agent. To evaluate the protective performance of silica SiO_2_ and zirconia ZrO_2_ nanoparticles in epoxy coatings, the coated samples were tested in a 3.5% NaCl solution. The experimental results clearly demonstrate a remarkable improvement in the ultimate tensile strength (UTS), yield strength (YS), and Elastic Modulus. In comparison to using SiO_2_ separately, the incorporation of both ZrO_2_ and SiO_2_ resulted in a substantial increase of 43.5% in UTS, 74.2% in YS, and 8.2% in Elastic Modulus. The corrosion test results revealed that the combination of DGEBA, SiO_2_, and ZrO_2_ significantly enhanced the anti-corrosion efficiency of the organic coatings. Both these pigments exhibited superior anti-corrosion effects and mechanical properties compared to conventional epoxy coatings, leading to a substantial increase in the anti-corrosion efficiency of the developed coating. This research focuses the potential of SiO_2_ and ZrO_2_ in hybrid combination for applications, where mechanical, corrosion and higher adhesion to the substrates are of prime importance.

## 1. Introduction

Epoxy coatings have emerged as a critical component in a myriad of industrial applications, owing to their superior mechanical properties [1], exceptional corrosion resistance [2], and excellent adhesion to a wide range of substrates [3]. These attributes make them an ideal choice for protective coatings in industries such as automotive [4], aerospace [5], marine [6], and construction [7]. However, the relentless evolution of these industries necessitates the continuous improvement of these coatings to meet the ever-increasing performance demands. One of the most promising strategies to achieve this is the incorporation of nanoparticles into the epoxy matrix [8,9], a technique that has the potential to significantly enhance the mechanical strength and anti-corrosion properties of the coatings [10,11].

In the realm of nanotechnology, silica (SiO_2_) and zirconia (ZrO_2_) nanoparticles have garnered considerable attention [12,13]. SiO_2_ nanoparticles are recognized for their high specific surface area and mechanical robustness, which can contribute to the hardness and durability of the coatings. On the other hand, ZrO_2_ nanoparticles are known for their high hardness, thermal stability, and excellent resistance to wear and corrosion, making them a valuable addition to enhance the overall performance of epoxy coatings [14,15].

In a study conducted by Allahverdi et al., an epoxy-based nanocomposite was prepared, utilizing Nano silica with an exceptionally high specific surface area as a reinforcing filler [16]. The mechanical properties of the nanocomposite, such as hardness and elastic modulus, were determined using nanoindentation, while its transparency was examined through transmission electron microscopy. The results demonstrated a significant enhancement in the mechanical properties of the nanocomposite, with increases of 26% and 21% in hardness and elastic modulus, respectively, when compared to neat epoxy. This was observed for resin filled with 5% Nano silica. Additionally, the glass transition temperature of the samples increased with the addition of silica nanoparticles, and an improvement in the thermal decomposition temperature of epoxy coating containing 5% Nano silica was reported [16].

Further research revealed that the corrosion resistance of the epoxy coating could be enhanced by incorporating 4–6% Nano-silica into the clear epoxy coating [17]. This was achieved by treating Nano-silica pigments with 3-Glycidoxypropyltrimethoxysilane (GPTMS). The Fourier transform infrared spectroscopy (FTIR) was employed to identify the grafting and incorporation of Nano-silica onto the epoxy matrix. Electrochemical impedance spectroscopy (EIS) confirmed that the incorporation of Nano-silica resulted in an enhancement in the corrosion resistance performance, with the final nanocomposite coatings exhibiting improved anti-corrosion efficiency [17].

In another study by Yang et al., the mechanical properties and thermal stability of epoxy-nanocomposite coating were improved by modifying it with different amounts of cube-like and rod-like CaC0_3_ nanoparticles, specifically, its mechanical properties with only 2% addition required [18]. The impact of incorporating silica Nano-particles onto epoxy coatings containing 2 wt.% of 130 nm silica particles was also investigated [19]. The silica particles were found to significantly enhance the hardness, increase surface roughness, and improve anticorrosive performance, as confirmed by electrochemical impedance spectroscopy [19].

A superhydrophobic polyester fabric has been fabricated by Sfameni et al. [20] by employing the eco-friendly sol-gel technique, along with hydrolysis and condensation of (3-glycidyloxypropyl) trimethoxy silane (GPTMS). It was crosslinked to different long alkyl-chain alkoxysilanes. The treated fabrics showed excellent super hydrophobicity. The application and utilization may be suitable and appropriate for sustainable water repellent and fluorine-free textile coatings for a wide range of sectors, such as technical textiles and protective workwear to automotive, from buildings to biomedical applications. The (3-glycidyloxypropyl) trimethoxy silane (G) has also been treated with different non-fluoro substances, such as triethoxy(ethyl)silane (C2), triethoxy(octyl)silane (C8), and hexadecyltrimethoxysilane (C16), in the presence of HCl and 1-methylimidazole as catalysts for the GPTMS epoxy ring opening, in order to prepare nanohybrid cross-linked polyalkylsiloxanes coatings. The developed films may be applied on polyester surfaces. Finally, fabricated micro- nanostructured hybrid coating was investigated and proposed with a possible mechanism of the super hydrophobic properties.

This research also involved the development of advanced and nanostructured epoxy adhesives by incorporating phenolic resin into epoxy using nanotechnology techniques [21]. This compensated for the low thermal stability of the epoxy. Rosa Medina et al. examined epoxy-ZrO_2_ nanocomposites prepared through mixing and dispersing techniques. They concluded that the addition of ZrO_2_ nanoparticles improved the tensile modulus, strength, and toughness of the epoxy matrix. Moreover, they reported that the nanoparticles induced specific fracture mechanisms, such as crack pinning and deflection, thus enhancing energy absorption before failure [22].

The innovative aspect of this work lies in the investigation of the synergistic effect of SiO_2_ and ZrO_2_ nanoparticles on the mechanical and corrosion protection properties of epoxy coatings. While previous studies have individually explored the incorporation of these nanoparticles into epoxy coatings, our research focuses on understanding the combined effect of these nanoparticles. We hypothesize that the simultaneous incorporation of SiO_2_ and ZrO_2_ nanoparticles can lead to cooperative effects, resulting in superior enhancements in the properties of epoxy coatings compared to the addition of each nanoparticle separately [23].

This paper presents an in-depth investigation into the synergistic effect of SiO_2_ and ZrO_2_ nanoparticles on the mechanical and corrosion protection properties of epoxy coatings. The study explores the potential enhancements in tensile strength, flexural strength, nanoindentation and corrosion resistance of epoxy adhesive coatings when these nanoparticles are incorporated. Furthermore, the research delves into the interaction between these nanoparticles within the epoxy matrix and how this interaction influences the overall performance of the coatings.

The findings of this research could provide valuable insights into the development of high-performance epoxy coatings for various industrial applications. By understanding the synergistic effects of SiO_2_ and ZrO_2_ nanoparticles, we can pave the way for the design of next-generation epoxy coatings with superior mechanical and anti-corrosion properties, thereby extending the lifespan and improving the reliability of coated structures in various industrial settings.

## 2. Materials and Methods

Epoxy resin Bisphenol A type and curing agent (Aradur D-450) were purchased from Hexion chemicals, Iserlohn and Huntsman Advance Materials, Deutschland, Germany. Acetone and Xylene were utilized as solvents and were purchased locally from Ideal Chemicals, Riyadh, KSA. Both silica (10–20 nm particle size) and zirconium (<100 nm) nanoparticles were acquired from Sigma-Aldrich under catalog numbers 637238 and 544760, respectively. All the materials purchased were used.

Formulations were prepared in a stoichiometric balanced amount of epoxy and hardener, where a fixed percentage of silica nanoparticles were added along with variable percentage of zirconium nanoparticles. A similar preparation procedure was followed as published elsewhere in our previous work [23], where the resin was initially placed in a beaker and diluted with xylene as a solvent to aid in mixing using a mechanical stirrer (Sheen S2 disperse master, Sheen instruments, Surrey, UK). In contrast, the SNPs were dispersed in acetone with the presence of silane using sonication to ensure proper mixing with the resin. The nanoparticle mixture was sonicated for 30 min. Subsequently, the dispersed nanoparticle solution was slowly added to the diluted epoxy resin while continuously stirring at 500 RPM using the mechanical stirrer. After the addition of the dispersed nanoparticles, the formulation underwent high-speed mechanical mixing at 5000 RPM for 45 min to facilitate dispersion and remove excess solvent. Once the mixing was complete, the stoichiometric amount of hardener was added to finalize the formulation. The coating formulating ingredients are briefly described in Table 1 along with the quantities.

DiscoverD8 from Bruker (Cu Kα, Bruker, Billerica, MA, USA) was utilized to perform X-ray diffraction (XRD) analysis to confirm the presence of nanoparticles in coatings and their effect on the morphology of prepared coatings. All the samples were scanned in the range of 10–80 degrees at the scan rate of 2°/min at room temperature. The thermal analysis was performed using Q600 from TA instruments (New Castle, DE, USA) to examine the degradation profile and effect of nanoparticles on decomposition temperature. The samples were heated from room temperature to 600 °C under nitrogen environment under ramp control with a ramping rate of 10°/min.

Conventional mechanical testing was performed with the Koenig pendulum (model 707/K, ASTM D-4366 [26]) and scratch resistance (model 705, ASTM D-7027 [27], Surrey, UK) from sheen instruments and impact resistance, in order to determine the mechanical properties of prepared coatings. The impact tester (model IG-1120, ASTM D-2794 [28], BYK, Columbia, SC, USA) was used to determine the impact resistance properties for prepared coatings on coated metal panel. The instrument comes with a fixed weight of 4lb inserted in a cylindrical tube with standard heights marked to evaluate the coating properties. The flat coated sample is placed right above the test die in such a manner that the indenter (or Punch) rests above the coating surface without exerting any force. The load in the cylindrical tube is raised up to a certain height and then we let it fall freely on the punch head, thereby exerting the force on the coated sample. The process of dropping the weight is repeated until we can ascertain the height at which the coating on the panel shows the sign of rupture or detachment from the panel. In order to provide consistency and reliability in the results, three different specimens are used, and similar steps are followed to drive the final impact resistance.

The coatings of the nanomechanical properties such as hardness and elastic modulus were also examined using nanotest platform from Micromaterials, Wrexham, UK. A Berkovich type indenter was used for this purpose. The samples were tested using a load control program with a constant loading of 1 mN/s until a maximum load of 250 mN is achieved. At 250 mN, the load was held for 60 s because of polymers viscoelastic nature (creep deformation) and then unloading was performed using the same rate until complete removal of load. At least 15 indentations were made at different locations and the results were shown as averaged.

Anticorrosion properties of the prepared coatings were analyzed in 3.5% NaCl solution for up to 30 days of exposure. The EIS experiments were performed using autolab PGSTAT 30 (Metrohm, Amsterdam, The Netherlands). The tests were performed with frequency scan within the range of 100,000 to 0.1 Hz by applying a ±5 mV amplitude sinusoidal wave perturbation.

## 3. Results and Discussion

### 3.1. Scanning Electron Microscope (SEM)

**Figure 1:**
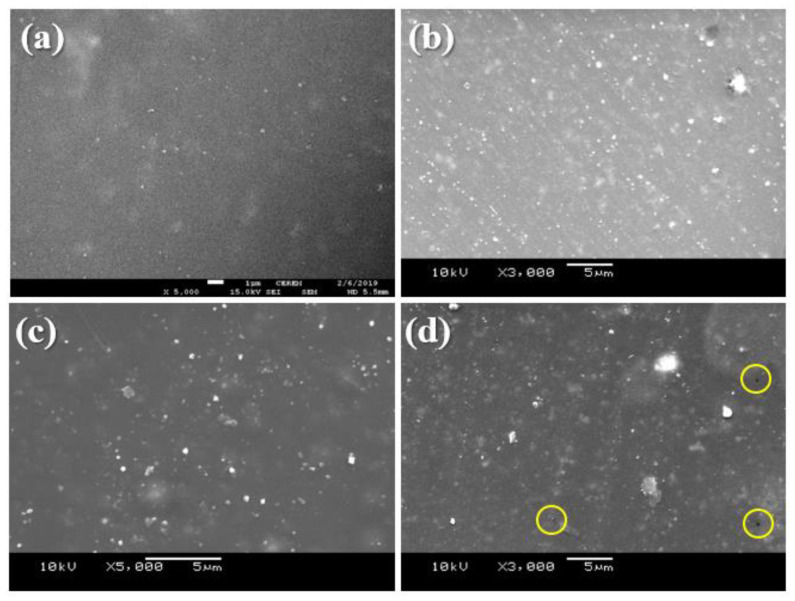
Scanning electron images (**a**) SN5 (**b**) SNZr-1 (**c**) SNZr-2 (**d**) SNZr-3.

The SEM images of the prepared coatings taken from the surface are shown in Figure 1. Image (a), representing SN5, describes the sample containing only with silica nanoparticles (0% zirconium), whereas images (b), (c) and (d) represent coatings with 1%, 2% and 3% zirconium nanoparticles along with silica. It can be seen clearly in Figure 1a, without the addition of zirconium nanoparticles, that coatings possess a homogenous and smooth surface. With the increasing percentage of ZrO_2_, the appearance started to change with the increase in the nanoparticles’ percentage; more bright white stops started to appear in the coatings. It was also observed that, with the addition of 1%, the surface was smooth and homogenous with an overall good distribution of nanoparticles. By increasing ZrO_2_ to 2%, the particles started to form aggregates; one more observed change in the surface was that the surface started to become a little uneven, and was not as smooth as 1% of nanoparticles. Through increments of ZrO_2_ nanoparticles until an achieved percentage of 3%, nanoparticles started to agglomerate, which can be seen in Figure 1d. With the particles starting to agglomerate, surface irregularities were more pronounced, and the coating surface became rougher and started to show defects in the first phase; these were pin holes and cracking, marked with the yellow circles. These defects are a result of a very high percentage of nanoparticles (5% SiO_2_ and 3% ZrO_2_), which leads to agglomeration because of the nanoparticles’ high surface area.

**Figure 2:**
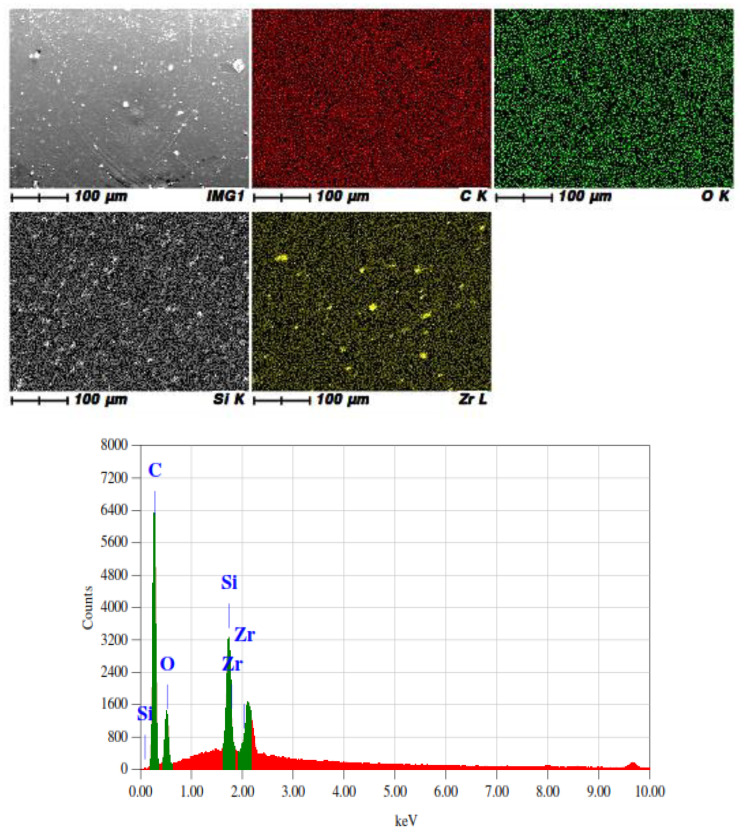
EDX mapping and spectrum of SNZr-3 coating sample.

To analyze the distribution of nanoparticles in coating, EDX was performed and results were mapped according to elements such as (C, O, Si and Zr); the results are shown in Figure 2. From the mapping, it is clear that silica nanoparticles were well distributed all over the coating, whereas the aggregates of ZrO_2_ nanoparticles can be seen in the Zr image. The overall distribution is good but the formation of agglomerates tends to reduce the coating properties.

### 3.2. Fourier Transform Infrared Spectroscopy (FTIR)

**Figure 3:**
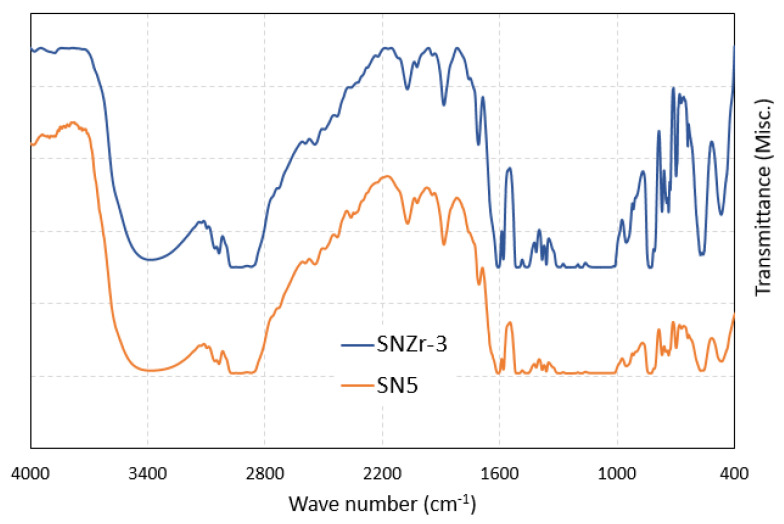
FTIR spectrum of epoxy with SiO_2_ (SN5) and Epoxy with SiO_2_/ZrO_2_ (SNZr-3).

Figure 3 shows the FTIR spectra of nano-modified epoxy coatings. FTIR analysis was performed in the range of 400 to 4000 cm^−1^. The broad peak in the region of (~3400 cm^−1^) corresponds to the OH group, and 1650 cm^−1^ and 830cm^−1^ corresponds to the NH band (primary amines) and epoxide ring, respectively [29]. Peaks in the range of 3000 cm^−1^ are because of C-H groups corresponding to (DGEBA) epoxy resin [30].

The characteristic peaks for SiO_2_/ZrO_2_ modified coatings are represented as per the following discussion. A broad peak in the range of 3200–3500 cm^−1^ are due to NH2 of the amine compound and OH-stretching; these bands also correspond to epoxy cross-linking reaction and ring opening. At 3038 cm^−1^, peak appearances correspond to C-H stretching. The interaction of hardener and epoxy was confirmed with the appearance of N-H band at 1605 cm^−1^. The appearance of peak at 467cm^−1^ is associated with Si-O-Si bending, thus confirming the presence of silica nanoparticles [31]. The peak appearance at band 753 cm^−1^ represents the Zr-O stretch vibration band, which appears after the addition of ZrO_2_ nanoparticles in the coating’s formulation [23].

### 3.3. Thermogravimetric Analysis (TGA)

**Figure 4:**
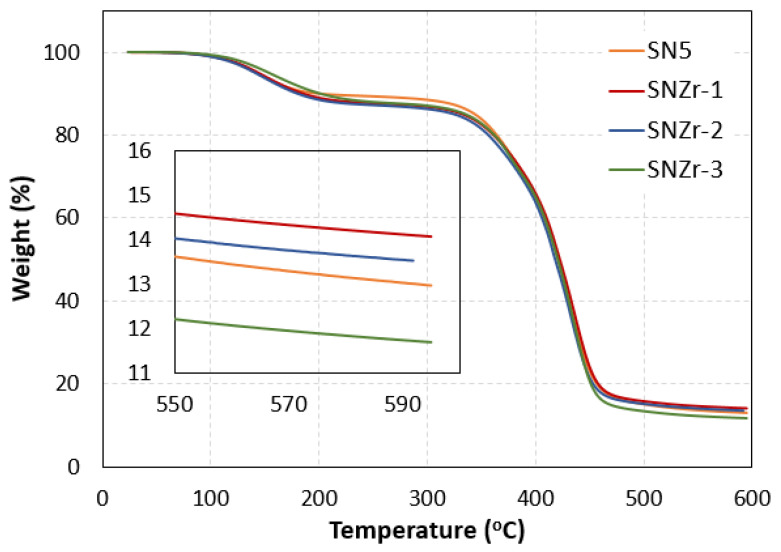
TGA cures of prepared epoxy coatings with different percentages of ZrO_2_.

The thermal properties of prepared coatings were tested using thermogravimetric analysis in nitrogen atmosphere; nitrogen was utilized to prevent the thermal oxidation of samples while heating at high temperatures. The TGA graphs of coatings prepared with varying percentages of nanoparticles are shown in Figure 4. From the presented graphs, it can be seen that all the samples undergo a two-step degradation process. The first stage of initial decomposition started after surpassing the 100 °C mark, followed by the main decomposition event starting from 300 °C to 500 °C. The major changes in the coating or evaluation of thermal properties is dependent on this second degradation stage [32,33].

The first stage of degradation is the decomposition of short chain molecules, residual reactants and trapped solvent and moisture molecules. It is approximately 15% of the total degradation profile. Second and main chain degradation started above 300C, which is the main epoxy chain degradation. Table 2 below describes the decomposition temperature at different intervals of degradation. The addition of the ZrO_2_ nanoparticle along with silica did not alter the thermal decomposition of the prepared coating. Approximate and similar decomposition temperatures were recorded for all the coatings. Additionally, the increase in nanoparticle percentages did not produce any effect on the thermal properties of coatings. The residual weight observed in TGA analysis can be affected by several factors, such as nanoparticle concentration, nature, matrix thermal stability, and experimental conditions. Interestingly, in certain cases, an increased nanoparticle content in the epoxy matrix can lead to a lower residual weight. This can be attributed to the enhanced thermal stability, improved char formation, improved dispersion, and enhanced crosslinking facilitated by the nanoparticles. Tuan Anh Nguyen et al. [34] also reported similar findings when investigating the use of TiO_2_ nanoparticles in an epoxy matrix.

### 3.4. Mechanical Properties and Nanoindentation

**Figure 5:**
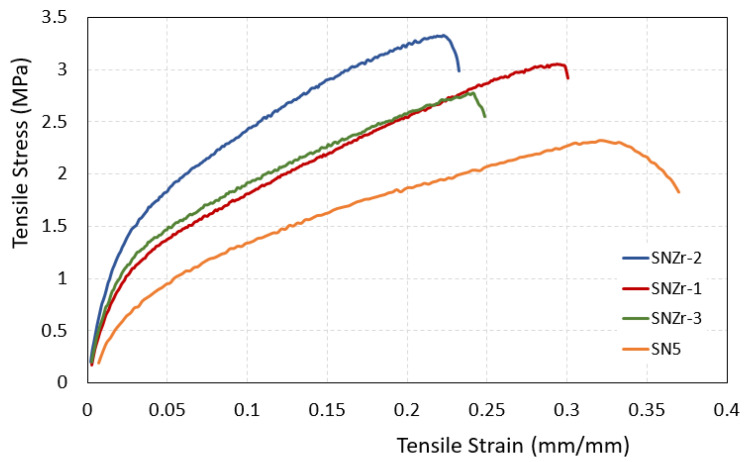
Tensile Stress–strain curves for silica and silica/ZrO_2_ filled epoxy coatings.

A comprehensive tensile test, using an Instron 150 kN machine, was conducted in accordance with ASTM D638-14 standard [35] on coatings containing silica and varying proportions of ZrO_2_ nanoparticles to assess their mechanical characteristics. The obtained stress–strain curves, as depicted in Figure 5 and Table 3, provide valuable insights. It is evident from the analysis that the coating labeled as SNZr-2 exhibited the highest ultimate strength, showcasing a remarkable improvement of 48.4% compared to other compositions. However, this enhancement in tensile strength comes at the expense of a decrease in the elongation property, as presented by the strain at break in Table 3, which is a typical trade-off in this particular scenario [36,37,38].

**Figure 6:**
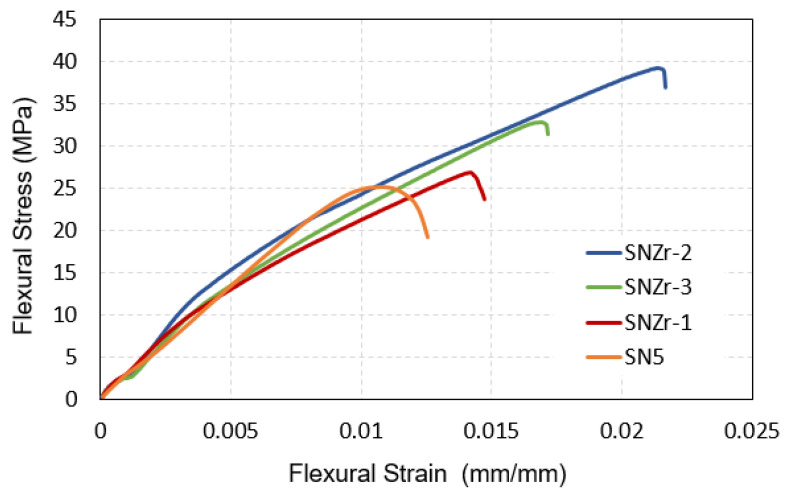
Flexure Stress–strain curves for silica and silica/ZrO_2_ filled epoxy coatings.

Figure 6 provides a comprehensive depiction of the conventional stress–strain curve, in addition to the alterations in the flexural strength and modulus, as determined through a three-point bending test in accordance with the ASTM D6412 standard [39]. The figure clearly demonstrates a notable enhancement in the area beneath the stress–strain curve, which is directly proportional to the increase in the content of ZrO_2_ nanoparticles.

This expansion in the area is not an isolated phenomenon. It is intrinsically linked with a marked improvement in two key parameters: the flexural modulus and the elongation at break. These improvements indicate a higher resistance to deformation and a greater ability to withstand stress without breaking, respectively.

The data analysis further reveals that the sample labeled as SNZr-2 stands out in terms of its mechanical properties. This sample exhibits a superior performance, which is in line with the results obtained from the tensile test. This consistency between different tests not only validates the robustness of the sample but also confirms the reliability of the testing methods used [36].

The mechanical properties of the coatings with silica, along with different percentages of ZrO_2_ nanoparticles, were studied with the help of pendulum hardness, scratch tester, impact resistance and nanoindentation. For consistency in the results, and by way of comparison, it is very important to measure the dry film thickness (DFT) of the coatings. For this purpose, sheen mini test 3100 was used to measure DFT after complete curing.

The obtained results for pendulum hardness, scratch and impact resistance are summarized in Table 4. The addition of ZrO_2_ nanoparticles, along with SiO_2_ nanoparticles, had a positive influence on the mechanical properties. The surface hardness (pendulum hardness) after incorporating ZrO_2_ nanoparticles show an enormous increase in the values, resulting in an increase of, at most, 44%. Similar results were seen with the scratch resistance, where the values obtained were at the maximum limit (10 kg) of our testing equipment. Impact strength, especially for coatings with 3% ZrO_2_ nanoparticles, show a decline because of particle agglomeration, as evident form the SEM images described above, while for the other added percentages, the obtained values are in very close range to SiO_2_-only coatings.

The effect of ZrO_2_ nanoparticles, along with SiO_2_ nanoparticles on the nanomechanical properties of coatings, were studied with the help of nanoindentation. The indentation of samples was carried out using a load control program, where the samples are loaded up to predefined maximum load, which is 250 mN in our case. The indentations on all the samples were performed using the Berkovich-type indenter, Micromaterials, Wrexham, UK.

**Figure 7:**
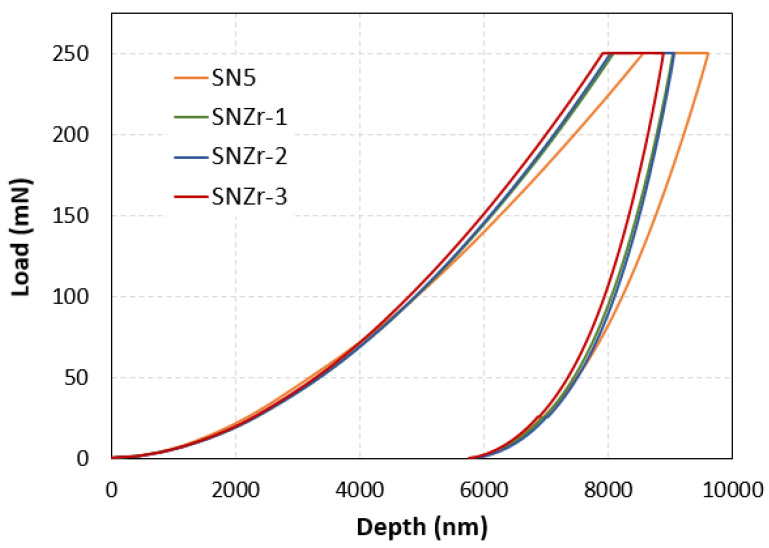
Load vs. depth curves for silica and silica/ZrO_2_ filled epoxy coatings.

The Indentation applied load vs. depth graphs for all the samples, and is shown in Figure 7. During the loading phase, as the load increased, its correspondence to depth increase was also recorded. In indentation, when the load is applied, the loading portion is representative of both elastic and plastic deformation behavior. The unloading portion of the graph represents the recovery of material after removal of load, hence the unloading portion is used to calculate the modulus of elasticity. Since polymeric materials are viscoelastic in nature, it is therefore important to apply a short-term creep in order to obtain accuracy in results. For this purpose, once the load reaches the maximum load, it is then held for a period of approximately 60s, this results in further increase in depth because of epoxy’s viscoelastic nature. This creep, if not applied, effects the unloading behavior of materials, which in turn has a negative influence on results [40].

All the prepared epoxy coatings were subjected to similar testing conditions. The load vs. depth curve, shown in Figure 7, describes that loading process was uniform without any cracking or non-uniformity. The addition of particles in the epoxy shows an improvement in the coatings’ properties, which is evident from shifting the maximum depth value at lower levels, toward the maximum applied load. This tells us that coatings are capable to hold higher loads [41]. The maximum depth values for samples with SiO_2_ only reached 8539 nm, with the incorporation of ZrO_2_ at 1%, 2% and 3%; the maximum depth values were reduced to 8052 nm, 8173 nm and 7892 nm, respectively.

The analysis of these graphs was performed using the machine software provided by micro materials. This analysis software works on the principle of the Oliver–Pharr model [42]. The mathematical expressions below are the representative of calculating the values of hardness and modulus.

(1) $$H=\frac{F_{max}}{A}$$

(2) $$E=\frac{1-{ʋ}_{s}^{2}}{\frac{1}{E_{r}}-\frac{1-{ʋ}_{i}^{2}}{E_{i}}}$$

where hardness is represented by H, F_max_ represents maximum load applied, A represents the projected area of contact at maximum load, E is the samples modulus, ʋ represents the materials poisons ratio (for polymer = 0.35), E_r_ is the reduced modulus obtained from indentation, E_i_ represents the modulus of indenter (1141 GPa for diamond) and ʋ_i_ is the poisons ratio of indenter (0.07 for diamond) [32,43].

The analysis results obtained are presented in Table 5. As seen from the table the addition of ZrO_2_ nanoparticles tends to increase both the hardness and modulus of the coating samples. With the increase in nanoparticles percentage properties are also improving. For silica only, coating hardness and elastic modulus obtained are 0.150 GPa and 3.284 GPa. With the addition of 2% ZrO_2_ nanoparticles, the properties significantly increased to 0.159 GPa and 3.553Gpa, which is an increment of approximately 6% in hardness values and 8% in modulus. Further increase in the percentages of ZrO_2_ also increased the hardness and modulus, but the author believes it is because of the indenter landing on the space where particles are in an aggregate form, which is evident in the SEM images (Section 3.1).

The increase in hardness is because of the addition of nanoparticles, which are the main cause of higher crosslinked density and reduction in free volume in complex epoxy crosslinked network. The reduction in free volume of the polymer matrix restricts the chain mobility; also, a reduction in free volume makes the material denser and more compact, resulting in higher properties. This dense and compact material then acts as a resistance to any external force applied, resulting in higher hardness [44,45].

### 3.5. X-ray Diffraction Analysis

X-ray diffraction analysis was performed for the samples of SN-5 and SNZr-1 to SNZR-3 coatings, in order to identify the peaks of SiO_2_ and ZrO_2_ after the absolute modification process and functionalization of nano pigments onto the epoxy matrix.

**Figure 8:**
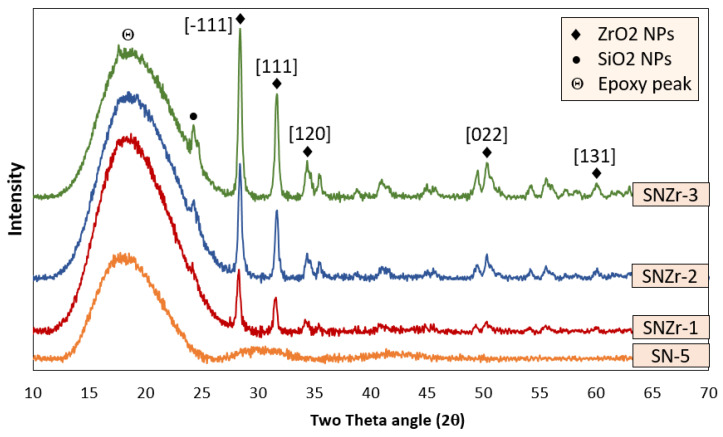
XRD Analysis of Nano-composite coatings (SN-5 Neat) and coatings (With ZrO_2_ Nano pigment).

The std. peaks of ZrO_2_ are shown in Figure 8 and the XRD pattern indicates the major peaks at 28, 34 degrees. The XRD pattern for the developed formulation SNZr1- SNZr-3 is also shown in Figure 6, where SN-5 peaks can be observed; the other peaks may consist of+ SiO_2_ and ZrO_2_. The addition of ZrO_2_ in the nanoparticle-incorporated coating (SNZr-1 to SNZR-3) is evident from the figure due to the presence of new peaks at 28–34 degrees. Also, the peaks of SNZr-1 to SNZR-3 formulation were observed to be slightly shifted when compared to the peaks of Neat (SN-5) formulation (with SiO_2_ nano-particles). This indicates that the ZrO_2_ was successfully incorporated onto the Epoxy matrix. The trends are almost the same regarding the epoxy peaks, while subsequent peaks of the nano-particles also appear at their respective locations. The intensity of the nano-particles peaks is quite less due to the concentration of the NPs being very low (1–3 wt%) in the epoxy matrix. This can be confirmed by the EDS pattern. The nature of the nano-particles is crystalline except for a few other nano-pigments [46].

### 3.6. Electrochemical Impedance Spectroscopy (EIS)

EIS provides information about the electrochemical processes occurring at the electrode-electrolyte interface. It can be used to analyze the kinetics of electrode reactions, determine the charge transfer resistance, measure the double-layer capacitance, and investigate the diffusion processes in the electrolyte [47]. The EIS method is widely used to study the corrosion and corrosion protection of metals and alloys when exposed to harsh environments. This technique provides valuable insights into the electrical properties of the materials, such as resistivity and capacitance, which are related to their corrosion behavior. EIS can be used to characterize the performance of coatings, inhibitors, and other protective measures, allowing researchers to optimize their formulations and develop more effective corrosion control strategies. Overall, EIS is a powerful tool for understanding and mitigating the effects of corrosion on a wide range of metallic systems [48,49,50]. EIS techniques were used to study the corrosion resistance of an epoxy coating containing SiO_2_ and ZrO_2_ nanoparticles. Increasing the concentration of ZrO_2_ nanoparticles from 1 wt% to 3 wt% showed a significant improvement in the corrosion resistance of the coating. The best corrosion resistance was for the 2 wt% ZrO_2_. This improvement was measured by an increase in the impedance of the coating in a 3.5% NaCl solution. The content of SiO_2_ was kept constant at 5 wt% throughout the study. These findings suggest that increasing the concentration of ZrO_2_ nanoparticles in epoxy coatings can improve their corrosion resistance.

**Figure 9:**
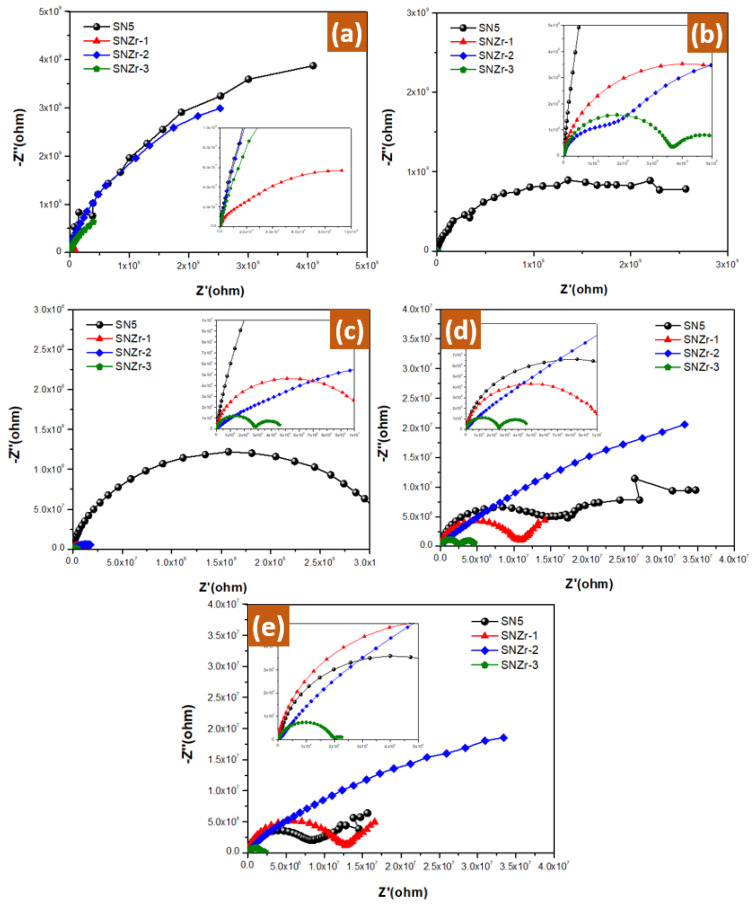
Nyquist plots of the fresh coatings after (**a**) 1 h, (**b**) 7 days, (**c**) 14 days, (**d**) 21 days and (**e**) 30 days’ immersion in a 3.5% NaCl solution.

Figure 9a displays the Nyquist plots for four different nanoparticle-incorporated epoxy coatings, SN5, SNZr-1, SNZr-2, and SNZr-3, after they were exposed to 3.5% NaCl solutions for 1 h. The plots indicate the impedance characteristics of each coating, with the SNZr-2 coating exhibiting the highest impedance values compared to the other coatings. These results suggest that SNZr-2 may be more effective in protecting against corrosion under the tested conditions. In order to assess how well the coatings were impregnated with ZrO_2_ and SiO_2_ nanoparticles able to resist corrosion, they were subjected to 3.5% NaCl exposure for up to 30 days with weekly intervals. The resulting Nyquist plots are presented in Figure 9b–e, with each figure representing a different period of exposure. These results provide valuable insight into the long-term effectiveness of the coatings and can help inform decisions about their potential use in various applications.

**Figure 10:**
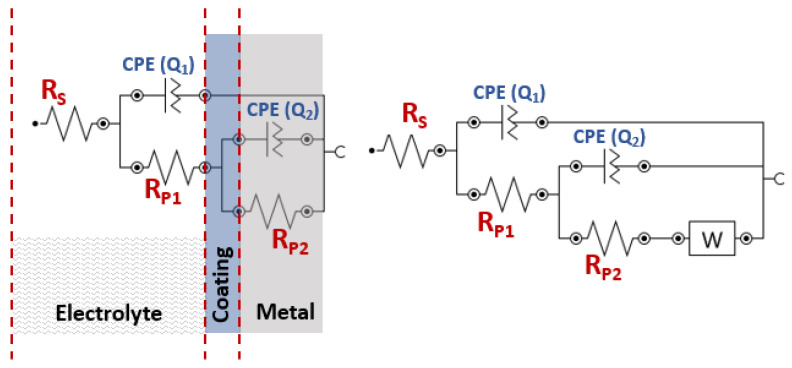
Equivalent electrical circuit models fitted to the obtained impedance.

A fitting of the obtained impedance data was performed, resulting in the equivalent circuit shown in Figure 10. This circuit model provides a representation of the electrical behavior of the system and allows for a better understanding of the underlying mechanisms at the interface. The equivalent circuit used to model the electrochemical behavior of epoxy coatings on metal surfaces consists of five to six elements: RS, CPE(Q1), RP1, CPE(Q2), RP2, and in some cases, W. RS represents the resistance of the solution, CPE(Q1), which represents constant phase element, and RP1 is the first polarization resistance between the epoxy coating surface and electrolyte. CPE(Q2), is the double layer capacitance, RP2, a second polarization resistance that could arise at the interface between the coating metal interphase, and W, a Warburg impedance.

**Figure 11:**
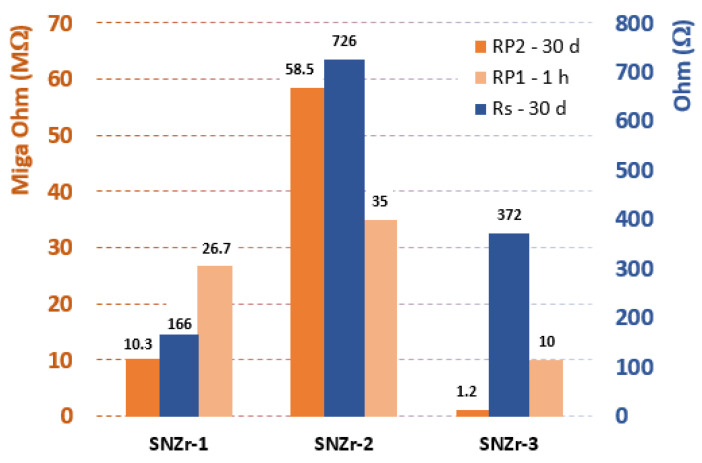
Overview of solution resistance (Rs) after 1 h of immersing time and both coating resistance (RP1) and metal interface resistance after 30 days of immersing time in aggressive medium of 3.5% NaCl.

The Nyquist spectra analysis revealed that an epoxy coating containing hybrid additives of 5 wt% SiO_2_ and 2 wt% ZrO_2_ demonstrated the best performance in terms of corrosion resistance. This supports the notion that a combination of ZrO_2_ and SiO_2_ content in nanoparticle-integrated coatings can improve corrosion resistance. The validation was reinforced with the EIS parameters presented in Table 6. These findings were achieved based on the readings listed in Table 6, where the values of RP1 and RP2 increase with the increase of ZrO_2_ nanoparticles in the epoxy coatings up to 2 wt%, as summarized in Figure 11.

In our prior studies [24], where we only used SiO_2_, it was concluded that the incorporation of SiO_2_ into epoxy coatings augmented their ability to withstand corrosion, specifically when the SiO_2_ concentration was augmented from 1% to 5%. Nonetheless, as the duration of exposure escalated, the coatings became more prone to degradation caused by water absorption. After confirming all results, it was determined that the presence of SiO_2_ had a positive impact on the traditional epoxy coating’s ability to resist corrosion. This result indicates that the inclusion of SiO_2_ could be a valuable addition to coatings designed to protect against corrosion. The overall outcome suggests that epoxy coatings with SiO_2_ have the potential to provide superior protection against corrosion. In the current study, another compound, ZrO_2_, was combined with SiO_2_ to improve the properties of the coating and its corrosion resistance. EIS data has confirmed that the passivation of coatings increases as the ZrO_2_ content increases in the epoxy matrix from 1% to 2%. This effect is due to the presence of both ZrO_2_ and SiO_2_ nanoparticles, which work together to create that synergistic effect [51]. This finding suggests that utilizing both nanoparticles in epoxy coatings could result in an improved corrosion resistance performance and longer durability.

The “n” component provides information about the purity of the capacitance in the circuit and can be used to identify the presence of other components, and also provides an indication of the porosity of a coating layer. “n” was found to range between 0.5 and 1 in the current study. This suggests that the coating layer is highly resistive and unlikely to develop many porosities. As “n” approaches 1, the semicircle in a capacitive impedance plot becomes more ideal, indicating a real capacitance. The decrease in Y_Q_ value is attributed to the corrosion resistance of the coatings against dissolution in chloride solution. The combination of CPE(Q1) and CPE(Q2) provide more insight into passivation and reduced porosity, which helps to prevent corrosion. The observed change in corrosion resistance over time, in comparison to the initial exposure period, is possibly due to the degradation of the coatings [41]. The impedance data collected indicate that the SNZr-2 coating exhibits the most exceptional resistance to corrosion, even when exposed to longer time periods. While the main reason behind the deterioration of the corrosion resistance of the SNZr-3 sample lies in the occurrence of agglomeration, whereas when the particles of the additive’s material gather around each other, this weakens their cohesion with the main matrix, causing the coating to lose its good anticorrosion and mechanical properties. This finding is consistent across all measured parameters and provides further evidence of the sample’s superior performance compared to other samples tested. Overall, this suggests that SNZr-2 may be an ideal material for use in applications where corrosion resistance is paramount.

## 4. Conclusions

This study successfully developed hybrid nano-composite epoxy formulations by leveraging the synergistic effects of SiO_2_ and ZrO_2_ nanoparticles. The addition of ZrO_2_ nanoparticles to the silica nanoparticles resulted in changes in the surface appearance of the coatings, with higher percentages leading to the formation of agglomerates, and surface irregularities. However, the distribution of silica nanoparticles remained well-maintained. Thermogravimetric analysis showed that the addition of ZrO_2_ nanoparticles did not significantly affect the thermal decomposition of the coatings, and the increase in nanoparticle percentage did not impact the thermal properties. The observed residual weight in the analysis could be influenced by factors such as nanoparticle concentration and matrix thermal stability. Tensile test results revealed that the coating labeled as SNZr-2, which contained silica and a specific proportion of ZrO_2_ nanoparticles, exhibited the highest ultimate strength compared to other compositions. However, this improvement in tensile strength came at the expense of a decrease in the elongation property, which is a common trade-off in such cases. The incorporation of ZrO_2_ nanoparticles in the coatings led to a notable enhancement in its mechanical properties, including increased area beneath the stress–strain curve and improved flexural modulus. The SNZr-2 sample consistently demonstrated superior mechanical performance across multiple tests.

Furthermore, the addition of ZrO_2_ nanoparticles to epoxy coatings improved their hardness and modulus values. This improvement can be attributed to increased cross-linked density, reduced free volume, and improved interfacial adhesion. These enhancements make the coatings more resistant to wear and deformation. Additionally, the addition of ZrO_2_ nanoparticles improved the corrosion resistance of the epoxy coatings. Coatings with 2 wt% ZrO_2_ nanoparticles exhibited the best performance, showing higher impedance values and improved passivation. The combination of ZrO_2_ and SiO_2_ nanoparticles resulted in a synergistic effect, enhancing the coatings’ corrosion protection. The SNZr-2 coating demonstrated the most exceptional resistance to corrosion. Overall, incorporating both ZrO_2_ and SiO_2_ nanoparticles in epoxy coatings can significantly enhance their mechanical properties and corrosion resistance of the fabricated coatings.

## Figures and Tables

**Table 1:** Formulation ingredients with quantity in the stoichiometric balance of the epoxy and the used hardener.

Sample	* Resin	Xylene (mL)	MIBK (mL)	SiO_2_ (wt%)	ZrO_2_ (wt%)	* Hardener	Ref.
SN5	83.34	10	10	5	0	16.66	[24,25]
Zr2	83.34	10	10	0	2	16.66	[23]
SNZr-1	83.34	10	10	5	1	16.66	Current study
SNZr-2	83.34	10	10	5	2	16.66	
SNZr-3	83.34	10	10	5	3	16.66	

* Stoichiometric balanced ratios in grams.

**Table 2:** Degradation temperatures recorded at various weight loss percentages for prepared coatings.

Sample	Degradation Temperature 15% Loss (°C)	Degradation Temperature 25% Loss (°C)	Degradation Temperature 50% Loss (°C)	Degradation Temperature 75% Loss (°C)	Ref.
SN5	344.10	377.80	420.90	447.03	[25]
Zr2	326.93	374.48	418.33	442.47	[23]
SNZr-1	332.10	377.02	421.54	447.84	Current study
SNZr-2	330.19	376.04	421.04	447.55	
SNZr-3	335.25	375.80	421.97	449.10	

**Table 3:** Numerical results for the ultimate tensile strength (UTS), yield strength (YS), and elongation in the tensile test.

Sample	Ultimate Tensile Strength (UTS), MPa	Yield Strength (YS), MPa	Strain at Break, (mm/mm)	Ref.
SN5	2.32	0.721	0.37	[25]
SNZr-1	3.05	0.968	0.30	Current study
SNZr-2	3.33	1.256	0.23	
SNZr-3	2.77	0.968	0.25	

**Table 4:** Mechanical properties obtained with different percentages of nanoparticles.

Sample	DFT µm	Pendulum Hardness	Scratch (Kg)	Impact (N/mm^2^)	Ref.
SN5	100 ± 10	118	9	0.8825	[25]
Zr2	100 ± 10	145	7.5	0.6067	[23]
SNZr-1	100 ± 10	170	10	0.8274	Current study
SNZr-2	100 ± 10	168	10	0.8274	
SNZr-3	100 ± 10	169	10	0.7171	

**Table 5:** Nanomechanical properties obtained with different percentages of nanoparticles.

Sample	Hardness (GPa)	Elastic Modulus (GPa)	Reference
SN5	0.150	3.284	[25]
Zr2	0.121	3.211	[23]
SNZr-1	0.157	3.451	Current study
SNZr-2	0.159	3.553	
SNZr-3	0.164	3.653	

**Table 6:** Electrochemical impedance spectroscopy (EIS) data of the SNZr coating samples after different immersion times in a 3.5% NaCl solution.

Coating	Time	EIS Parameters							
		R_S_ (Ω)	CPE (Q_1_)		R_P1_ (MΩ)	CPE (Q_2_)		R_P2_ (MΩ)	W (nMho)
			nMho	n		nMho	n		
SNZr-1	1 h	562	0.938	0.971	26.7	4.48	0.682	160	-
	7 d	258	1.15	0.967	1.93	2.51	0.667	6.48	994
	14 d	231	1.13	0.968	2.27	1.78	0.694	8.60	675
	21 d	151	1.11	0.971	1.55	1.80	0.692	8.53	710
	30 d	166	1.13	0.971	1.64	1.56	0.705	10.3	614
SNZr-2	1 h	321	1.01	0.973	35.0	0.636	0.647	11 × 10^3^	-
	7 d	1180	1.91	0.927	2.10	26.9	0.759	10.4	-
	14 d	200	0.848	0.999	68.5	86.3	0.484	28.9	-
	21 d	2470	2.91	0.894	0.0861	70.1	0.540	82.5	-
	30 d	726	1.94	0.930	0.0896	63.2	0.587	58.5	-
SNZr-3	1 h	85.1	0.806	0.982	10.0	0.999	0.623	3.37 × 10^3^	-
	7 d	1800	1.61	0.928	3.58	829	0.772	2.22	-
	14 d	1620	1.55	0.932	2.75	1220	0.774	2.12	-
	21 d	1480	1.40	0.940	2.47	1210	0.783	2.57	-
	30 d	372	1.41	0.900	1.60	1090	0.512	1.20	-

## Data Availability

The data presented in this study are available from the corresponding author upon reasonable request.
